# Intracarotid Infusion of Redox-Active Manganese Porphyrin, MnTnBuOE-2-PyP^5+^, following Reperfusion Improves Long-Term, 28-Day Post-Stroke Outcomes in Rats

**DOI:** 10.3390/antiox12101861

**Published:** 2023-10-13

**Authors:** Xuan Li, Weina Duan, Li Du, Dongmei Chu, Peng Wang, Zhong Yang, Xingguang Qu, Zhenxing Yang, Ines Batinic-Haberle, Ivan Spasojevic, David S. Warner, James D. Crapo, Miriam M. Treggiari, Huaxin Sheng

**Affiliations:** 1 Multidisciplinary Neuroprotection Laboratories, Center of Perioperative Organ Protection, Department of Anesthesiology, Duke University Medical Center, Durham, NC 27710, USA; lixuan@hrbmu.edu.cn (X.L.); duanweina@whu.edu.cn (W.D.); duliwh@whu.edu.cn (L.D.); chudongmei1125@126.com (D.C.); kylinwp@163.com (P.W.); yangzhong305@126.com (Z.Y.); quxingguang1961@ctgu.edu.cn (X.Q.); david.warner@duke.edu (D.S.W.); miriam.treggiari@duke.edu (M.M.T.); 2 Department of Neurosurgery, Duke University Medical Center, Durham, NC 27710, USA; neuroyzx@whu.edu.cn; 3 Department of Radiation Oncology, Duke University Medical Center, Durham, NC 27710, USA; ibatinic@duke.edu; 4 Pharmacokinetics and Pharmacodynamics Core, Duke Cancer Institute, and Department of Medicine, Duke University Medical Center, Durham, NC 27710, USA; 5 Department of Neurobiology, Duke University Medical Center, Durham, NC 27710, USA; 6 BioMimetix JV LLC, Greenwood Village, CO 80111, USA; james.crapo@bmxpharma.com

**Keywords:** ischemic stroke, thrombectomy, BMX-001, manganese porphyrin, intracarotid, long-term, 28-day post-stroke outcome

## Abstract

Endovascular mechanical thrombectomy, combined with a tissue plasminogen activator (t-PA), is efficacious as a standard care for qualifying ischemic stroke patients. However, > 50% of thrombectomy patients still have poor outcomes. Manganese porphyrins, commonly known as mimics of superoxide dismutases, are potent redox-active catalytic compounds that decrease oxidative/nitrosative stress and in turn decrease inflammatory responses, mitigating therefore the secondary injury of the ischemic brain. This study investigates the effect of intracarotid MnTnBuOE-2-PyP^5+^ (BMX-001) administration on long-term, 28-day post-stroke recovery in a clinically relevant setting. The 90 min of transient middle cerebral artery occlusion was performed in young, aged, male, female, and spontaneous hypertension rats. All physiological parameters, including blood pressure, blood gas, glucose, and temperature, were well controlled during ischemia. Either BMX-001 or a vehicle solution was infused through the carotid artery immediately after the removal of filament, mimicking endovascular thrombectomy, and was followed by 7 days of subcutaneous injection. Neurologic deficits and infarct volume were assessed at 28 days in a blinded manner. The effects of BMX-001 on the carotid arterial wall and blood–brain barrier permeability and its interaction with t-PA were assessed in normal rats. There were no intra-group differences in physiological variables. BMX-001-treated stroke rats regained body weight earlier, performed better in behavioral tests, and had smaller brain infarct size compared to the vehicle-treated group. No vascular wall damage and blood–brain barrier permeability changes were detected after the BMX-001 infusion. There was no drug interaction between BMX-001 and t-PA. Intracarotid BMX-001 infusion was safe, and it significantly improved stroke outcomes in rats. These findings indicate that BMX-001 is a candidate drug as an adjunct treatment for thrombectomy procedure to further improve the neurologic outcomes of thrombectomy patients. This study warrants further clinical investigation of BMX-001 as a new stroke therapy.

## 1. Introduction

Stroke is a leading cause of death and disability in the United States. Nearly 800,000 people suffer from this disease each year, and 87% of these patients are ischemic in nature [1]. When a cerebral artery is blocked by a blood clot, it will immediately lead to neurologic and functional deficits [2]. The brain tissue within the arterial territory is metabolically compromised at the beginning; it is still salvageable if reperfusion can be achieved within a limited period of time [3,4,5]. The early restoration of the blood flow is critical for the treatment of stroke patients. Intravenous tissue plasminogen activator (t-PA) was approved by the FDA as a standard thrombolytic therapy [6,7,8], and endovascular mechanical thrombectomy also became popular as an effective recanalization procedure [9,10,11,12,13,14,15], significantly improving clinical outcomes. However, there are some shortcomings of these treatments, including the narrow therapeutic time window for t-PA of 4.5 h after the onset of a stroke, which limits its use to <10% of patients, [16,17] and more than 50% of thrombectomy patients still do not recover functional independence [18,19,20]. Therefore, there is an urgent need for the development of a new compound to overcome these obstacles.

Manganese (III) *ortho* pentacationic, *N*-alkyl- or *N*-alkoxyalkylpyridylporphyrins and *N*,*N’*-alkylimidazolylporphyrins are potent redox-active compounds with exceptional superoxide dismutase activity. Several animal studies have demonstrated that such compounds protect the brain against ischemic and reperfusion injuries [21,22,23]. The efficacy was present even when Mn porphyrin was given 6 h after reperfusion [22]. A long-term effect was achieved with a 7-day treatment [21]. Ischemia and reperfusion induced the formation of reactive oxygen and nitrogen species, which, through the activation of the NF-κB transcription factor, exacerbated brain inflammation. Such events were attenuated by several Mn porphyrins [21,22,23]. BMX-001 (MnTnBuOE-2-PyP^5+^) is a manganese porphyrin currently evaluated in Phase II clinical trials on patients bearing high-grade glioma, multiple brain metastases, anal cancer, and head and neck cancer [23,24]. Several studies have already shown the ability of BMX-001 to inhibit NF-κB while activating Nrf2 transcription factors. The latter transcription factor controls endogenous antioxidative defenses. Both NF-κB and Nrf2 contribute to the therapeutic efficacy of BMX-001 [23,24]. A selective, direct administration of BMX-001 at the site of the stroke has not been previously evaluated. This study was designed to investigate the 28-day post-stroke effect of intracarotid BMX-001 infusion in a rat model of ischemic stroke. This is a potentially highly translatable model, because a catheter is in place for thrombectomy patients, and BMX-001 could serve as an adjunct to thrombectomy to enhance the efficacy of stroke treatment.

## 2. Materials and Methods

The following experiments were approved by the Duke University Animal Care and Use Committee. The care and handling of the animals were in accordance with the National Institutes of Health guidelines. Male Wistar rats (Hsd:WI, 250–275 g) were purchased from Envigo (Indianapolis, IN, USA) and housed at the Duke vivarium with free access to food and water and a 12 h light/12 h dark cycle. Room temperature and humidity were well controlled.

*Ischemic Stroke Model.* The rats were fasted overnight for glucose control. On the surgery day, they were anesthetized with 5% isoflurane in 30% oxygen balanced with nitrogen. The trachea was orally intubated, and both lungs were mechanically ventilated to maintain normocapnia. Isoflurane was reduced to 1.5% during the surgical procedure. A 22-gauge needle thermistor was percutaneously inserted beneath the temporalis muscle adjacent to the skull. Pericranial temperature was maintained at 37.0 °C ± 0.2 °C with a surface heat lamp. The tail artery was cannulated with PE 50 tubing (BD Intramedic^TM^ Polyethylene Tubing, Sparks, MD, USA), and arterial blood pressure was continuously monitored. A dose of 50 IU heparin was administered via arterial line to prevent intra-arterial thrombosis. Arterial blood samples were collected pre, during, and after ischemia for blood gas, glucose, and hematocrit measurements.

Ischemic stroke was induced by transiently occluding the middle cerebral artery (MCAO), as previously reported [25]. In brief, a midline cervical incision was cut, and the right common carotid artery (CCA) was identified. The external (ECA) and internal carotid arteries were dissected. The distal external carotid artery and its superior thyroid branch were isolated, ligated, and divided. The internal carotid artery (ICA) was dissected distally until the origin of the pterygopalatine artery was visualized. After surgical preparation, a 10 min interval was allowed for physiologic stabilization. Then, the internal and common carotid arteries were temporally blocked, a small incision was cut on the ECA stump, and a silicone-coated 4-0 nylon monofilament (Doccol MCAO suture, 403745) was inserted through the ECA and passed distally into the ICA until a slight resistance was felt; then, it was secured using a silk suture. The common carotid artery was re-opened, and a timer was started at the onset of cerebral ischemia.

After 90 min MCAO, the filament was removed and a PE-10 tube (BD Intramedic^TM^ Polyethylene Tubing, Sparks, MD, USA) was inserted into the ICA beyond the origin of the pterygopalatine artery. The vehicle or BMX-001 (30–50 µg/kg) solution (1 µL/g for both solutions, 250 µL for the 250 g rat) was slowly infused for 5 min using a syringe pump. At the completion of the intra-carotid infusion, the PE-10 tube was removed, and the ECA stump was permanently ligated. The tail artery catheter was removed, and both the neck incision and tail incision were infiltrated with bupivacaine and closed with sutures. Isoflurane was then discontinued. The rats were disconnected from the ventilator when they had spontaneous respiration and kept in a recovery cage. The tracheas were extubated after the recovery of the righting reflex. They remained in an O_2_ enriched environment (30% O_2_) for 1 h and were then returned to their home cage.

*Neurologic score.* Post-stroke neurologic deficits were assessed at 7 and 28 days using a standardized neurologic scoring system [22]. General status, simple motor deficit, complex motor deficit, and sensory deficit were evaluated, including the ability of walking on a beam and climbing on the vertical screen. The score each animal received was the sum of all four individual scores (0–48, 0 = normal, and 48 = the worst score). This scoring system has been shown to correlate well with cerebral infarct volume measurements [22].

*Cerebral infarct volume measurement.* After the completion of functional tests, the animals were weighed, anesthetized with isoflurane, and decapitated. The brains were harvested, frozen at −20 °C in 2-methylbutane on dry ice and stored at −80 °C in a freezer. Serial quadruplicate 20-µm-thick coronal sections were cut using a Leica cryostat and mounted on the slides at a 720-µm interval over the rostral–caudal extent of the brain. The sections were dried on a slide heater at 36 °C and stained with hematoxylin and eosin. A section from each 800-µm interval was digitized with a video camera controlled by an image analyzer (MCID, the MicroComputer Imaging Device, Imaging Research Inc, St. Catharines, Ontario, Canada). The image of each section was stored as a 1280 x 960-pixel matrix and displayed on a computer monitor. With the observer blinded to the experimental condition, regions of interest (ROI) were outlined individually using an operator-controlled cursor, including the noninfarcted ipsilateral cerebral cortex, noninfarcted ipsilateral subcortex, contralateral cerebral cortex, and contralateral subcortex. The area in each ROI (mm^2^) was determined by the automated counting of calibrated pixels, and the volume (mm^3^) was computed by a known interval between sections over the rostral–caudal extent of the brain. Ischemic tissue volume was calculated by subtracting ipsilateral noninfarcted tissue volume from the corresponding contralateral tissue volume.

### 2.1. Experiment 1: Effect of BMX-001 on Acute Stroke Outcomes in Young Male Rats

A total of 30 young male Wistar rats (250–275 g, 9 weeks old) were subjected to 90 min MCAO and then randomly assigned to the vehicle (*n* = 16) or BMX-001 treatment group (*n* = 14). The BMX-001 solution was prepared in saline, which served as vehicle. A bolus dose was given through the carotid artery immediately after reperfusion, followed by subsequent maintenance doses given through the subcutaneous route for one week. The doses were determined from the pilot experiment, showing that a 30 µg/kg BMX-001 intra-carotid infusion over 5 min or a 225 µg/kg subcutaneous injection twice daily did not cause a large blood pressure drop. Thus, these stroke rats received 30 µg/kg BMX-001 or vehicle intra-carotid infusions and subsequent 225 µg/kg BMX-001 or vehicle subcutaneous injections twice per day for 7 days. The animals were weighed and fed with soft food daily. Neurological deficits and infarct volumes were examined at 7 days post-stroke.

### 2.2. Experiment 2: Effect of BMX-001 on 28-Day Post-Stroke Outcomes in Young Female Rats

A total of 30 young female Wistar rats (225–250 g, 9 weeks old) were subjected to 90 min MCAO and randomly assigned to the vehicle (*n* = 15) and BMX-001 treatment group (*n* = 15). They received 7 days of treatment using the protocol described in Experiment 1. The animals were weighed and fed with soft food daily during the first week. Neurologic scores were evaluated at 7 and 28 days. Infarct volumes were measured at 28 days.

### 2.3. Experiment 3: Effect of BMX-001 Treatment Duration on 28-Day Post-Stroke Outcomes in Young Male Rats

A total of 57 young male Wistar rats (250–275 g, 9 weeks old) were subjected to 90 min MCAO and then randomly assigned to the following 3 groups: Group 1: Vehicle intra-carotid bolus plus 28 days of vehicle maintenance doses (*n* = 19); Group 2: BMX-001 intra-carotid bolus plus 7 days of maintenance doses followed by 21 days of vehicle maintenance doses (*n* = 19); and Group 3: BMX-001 intra-carotid bolus plus 28 days of maintenance doses (*n* = 19). The dose of the intra-carotid BMX-001 bolus was increased to 50 µg/kg. Thus, the stroke animals were treated with a vehicle or 50 µg/kg BMX-001 intra-carotid infusion followed by 28 days of subcutaneous vehicle or 225 µg/kg BMX-001 injections for 7 or 28 days. As a new batch of GMP synthesis/scale-up BMX-001 was received, we re-evaluated the effect of intra-carotid infusion on blood pressure. The animals were weighed and fed with soft food daily during the first week. Neurologic scores were evaluated at 7 and 28 days. Infarct volumes were measured at 28 days.

### 2.4. Experiment 4: Effects of BMX-001 on Stroke Outcomes in Young Spontaneous Hypertension Rats

A total of 30 young male spontaneous hypertension rats (SHR rats, 225–250 g, 8 weeks old) were subjected to 90 min MCAO and randomly assigned to the vehicle (*n* = 15) or BMX-001 treatment (*n* = 15) group as described in Group 1 and Group 2 of Experiment 3. The animals were weighed and fed with soft food daily during the first week. Neurologic scores were evaluated at 7 and 28 days. Infarct volumes were measured at 28 days.

### 2.5. Experiment 5: Effect of BMX-001 on Stroke Outcomes in Aged Female Rats

A total of 34 aged female Fisher 344 rats (220–250 g, 22 months old) were subjected to 90 min MCAO and randomly assigned to the vehicle (*n* = 17) or BMX-001 (*n* = 17) group. The treatment and outcome measures were the same as in experiment 4.

### 2.6. Experiment 6: BMX-001 Brain Concentration Measurement

Young male Wistar rats (250–275 g, 9 weeks old, *n* = 4) were subjected to a 90 min MCAO and 30 µg/kg BMX-001 intra-carotid infusion following reperfusion. Blood and brain samples were harvested at 30 min after BMX-001 infusion, stored in a −80°C freezer, and measured using LCMS/MS, as reported earlier [26]. In brief, the BMX-001 was ion-paired with heptafluorobutyric acid and extracted from samples by chloroform/isopropanol (4/1). After evaporation to dryness, the residue was reconstituted in mobile phase and injected into LC/MS/MS system, Agilent 1100/1200 LC (Santa Clara, CA 95051, USA) and AB/SCIEX API-5500 MS/MS (Framingham, MA 01701, USA). Deuterated analog, BMX-001-d8 was used as internal standard.

### 2.7. Experiment 7: Effect of BMX-001 Infused via Carotid Artery on Blood–Brain Barrier Permeability and the Vascular Endothelial Wall

Normal male Wistar rats (250–275 g, 9 weeks old) received 30 µg/kg BMX-001 (*n* = 4), the same amount of saline (*n* = 4), or 5 mL/kg 20% mannitol (*n* = 4) through the carotid artery. The rats received a 100 µL 2% Evans blue intravenous infusion 30 min later and were then intracardially perfused with 100 mL normal saline at 30 min following infusion. Their brains were harvested, and the Evans blue content was measured spectrophotometrically.

Additionally, normal Wistar rats (250–275 g, 9 weeks old) received 30 µg/kg BMX-001 (*n* = 4), the same volume of saline (*n* = 4), or 50 µL 62.5 mg/mL Solu-Medrol (*n* = 4) through the carotid artery. The rats were recovered for 3 days and were perfused intracardially with normal saline followed by 10% formalin. Their brains were paraffin embedded, cut, and stained with hematoxylin and eosin. Vascular histologic damage was assessed.

### 2.8. Experiment 8: Interaction of BMX-001 with t-PA

Fresh arterial blood samples were collected from young male rats (250–275 g, 9-weeks-old), distributed into 21 pre-weighed centrifuge tubes, and rested at 37 °C for 3 h until the blood clotted. The liquid portion on the top of the clot was removed from these tubes and then the tubes with the blood clots were weighed. The weight of the blood clot was calculated (=the tube with blood clot − pre-weighed tube). Then, 1 mL saline, t-PA (1 mg/mL) or t-PA (1 mg/mL) plus BMX-001 (50 µg/mL) were added (*n* = 7 for each group) and placed in a 37 °C dry incubator for 3 h. The liquids were removed from the tubes. The tubes with remaining blood clots were weighed again. The percentage of the blood clot dissolved by t-PA was calculated.

For all experiments, the outcome measures were performed by trained laboratory personnel who were blinded to the group assignment.

### 2.9. Statistical Analysis

Data are expressed as mean ± SD, unless otherwise specified. We used unpaired Student *t*-test or one-way ANOVA for experiments with more than two groups. Since neurologic scores are nonparametric data, their summaries are expressed as median ± IQR, and analyzed using the Mann–Whitney test. All statistical analyses were performed using Prism 6 software (GraphPad Software Inc, San Diego, CA, USA), and a *p*-value < 0.05 was considered statistically significant.

## 3. Results

### 3.1. Experiment 1: BMX-001 Provided Acute Protection in Young Male Stroke Rats

A total of 30 young male rats were subjected to 90 min MCAO. One died during surgery due to bleeding from the carotid artery, and one vehicle rat died 24 h after stroke. Intracranial hemorrhage was not found. Three rats, including two vehicle rats and one BMX-001 rat, did not circle to the left after recovering from anesthesia. Thus, 5 rats were excluded from this study. There were 12 vehicle and 13 BMX-001 rats analyzed for outcomes.

**Figure 1:**
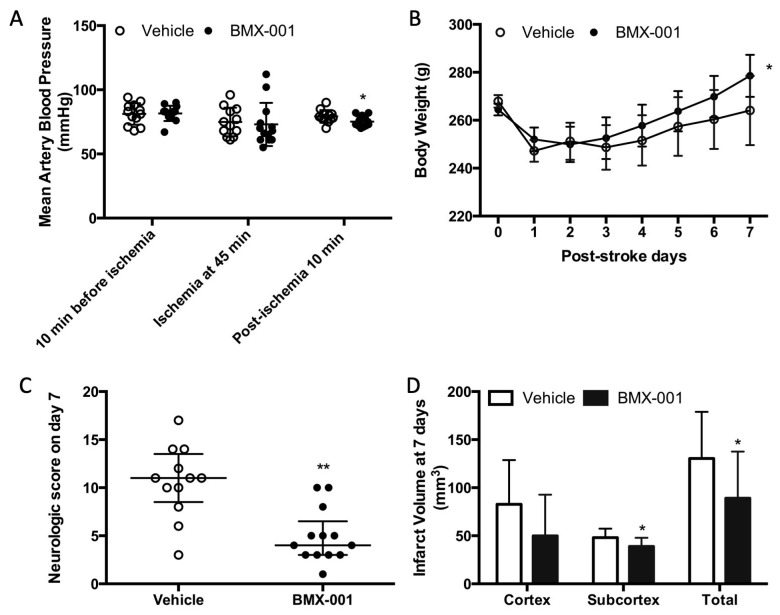
Treatment with BMX-001 improved the post-stroke outcome in young male Wistar rats. Animals had a 90 min MCAO and received vehicle or BMX-001 treatment for 7 days. Peri-operative blood pressure was measured during surgery, and the BMX-001 group had a slight low blood pressure at 5 min after treatment (**A**). Ischemic injury induced significant body weight loss at 24 h post-stroke (**B**), and the BMX-001 treatment promoted post-stroke body weight recovery (*p* = 0.02 in BMX-001 and *p* = 0.27 in vehicle; body weight at 7 days vs. body weight at 24 h). Both neurologic scores (**C**) and infarct volume (**D**) were significantly improved with the BMX-001 treatment. Neurologic scores were expressed as median ± IQR, and other data were expressed as mean ± SD, * *p* < 0.05, ** *p* < 0.01.

Perioperative blood pressures (10 min before ischemia, 45 min of ischemia, and 10 min post-ischemia) were 81 ± 8 mmHg, 75 ± 11 mmHg, and 79 ± 5 mmHg in the vehicle and 82 ± 6 mmHg, 73 ± 16 mmHg, and 75 ± 4 mmHg in the BMX-001 group. There was no intra-group blood pressure difference at 10 min before ischemia and 45 min after the onset of ischemia. However, we found that the blood pressure was statistically—but not clinically—significantly lower after the BMX-001 infusion (79 ± 5 mmHg in the vehicle and 75 ± 4 in BMX-001, *p* = 0.04, Figure 1A).

The animals in both groups had a significant body weight loss at 24 h post-stroke (247 ± 15 g in vehicle, *p* < 0.001 vs. baseline 267 ± 9 g, and 252 ± 9 g in BMX-001, *p* < 0.001 vs. baseline 265 ± 9 g). Body weight loss was slowly recovered in the vehicle-treated rats (264 ± 14 at 7 days, *p* = 0.278 vs. 247 ± 15 g at 24 h). The BMX-001 treatment promoted body weight recovery (279 ± 9 g at 7 days, *p* = 0.02 vs. 252 ± 9 g at 24 h, Figure 1B).

Neurologic scores on day 7 post-stroke were 11 ± 5 in the vehicle-treated animals and 4 ± 3.5 in BMX-001-treated animals (*p* < 0.01, Figure 1C), indicating significant functional improvement in the BMX-001 group. The vehicle group had a large brain infarct (total infarct 130.354 ± 48.591 mm^3^, cortex infarct 82.670 ± 46.134 mm^3^, and subcortex infarct 48.108 ± 9.256 mm^3^), and the BMX-001 group had a smaller infarct size (total infarct 89.097 ± 48.424 mm^3^, *p* = 0.04 vs. vehicle; cortex infarct 49.864 ± 42.899 mm^3^, *p* = 0.07 vs. vehicle; subcortex infarct 38.964 ± 8.892 mm^3^, *p* = 0.02 vs. vehicle, Figure 1D).

### 3.2. Experiment 2: BMX-001 Reduced Neurologic Deficits in 28-Day Post-Stroke Survived Young Female Rats

**Figure 2:**
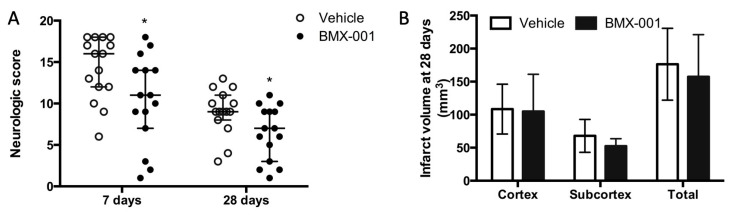
Treatment with BMX-001 improved the 28-day post-stroke outcome in female rats. The animals had a 90 min MCAO and received vehicle or BMX-001 treatment for 7 days. Neurologic scores were evaluated at 7 days and 28 days after stroke (**A**), and infarct volumes were measured at 28 days (**B**). The BMX-001-treated group had a better neurologic score at 7 days (*p* = 0.04) and 28 days (*p* = 0.04) compared to the vehicle group. Infarct volume was smaller in the BMX-001-treated group and no intragroup difference was detected. Neurologic scores were expressed as median ± IQR, and the infarct volume was expressed as mean ± SD, * *p* < 0.05.

A total of 30 female rats were subjected to 90 min MCAO, and all of them survived for 28 days. Although these female rats were of the same age as the males, they were smaller in appearance size. We used the same size of filament for both male and female rats, which induced more severe ischemia in female rats compared to male rats, probably due to the small ICA diameter in female rats. Neurologic scores at 7 days were 16 ± 6 in the female vehicle group and 11 ± 5 in the male vehicle rats. The BMX-001 treatment improved post-stroke neurologic deficits at both 7 days and 28 days (Figure 2A, 7 days, 16 ± 6 in vehicle, 11 ± 7 in BMX-001, *p* = 0.04; 28 days, 9 ± 3 in vehicle, 7 ± 6 in BMX-001, *p* = 0.04). Although BMX-001-treated rats had a small infarct volume compared to the vehicle-treated rats, these differences were not statistically significant (Figure 2B, 157.378 ± 63.699 mm^3^ in BMX-001 vs. 176.334 ± 54.432 mm^3^ in vehicle, *p* = 0.38). It is possible that the doses of BMX-001 were not sufficient for rescuing the brain tissue that underwent severe ischemia.

### 3.3. Experiment 3: Treatment with BMX-001 for 7 Days Provides 28-Day Post-Stroke Protection in Young Male Rats

The results for the perioperative physiological parameters, including blood pressure, blood gas, glucose, and hematocrit, were controlled at similar levels in the 57 rats subjected to 90 min MCAO and are shown in Table 1. There was no intra-group difference.

**Figure 3:**
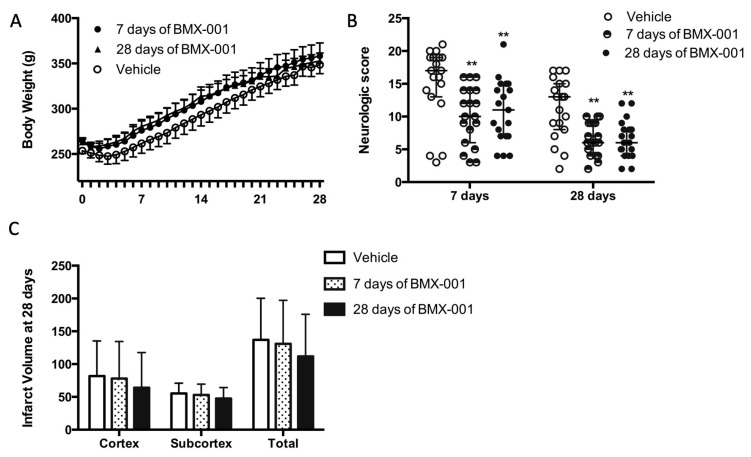
Functional recovery was improved after 7 days of BMX-001 treatment on day 28 in male stroke rats. The animals had a 90 min MCAO and then received an intracarotid bolus infusion of vehicle or BMX-001 treatment, followed by subcutaneous maintenance doses for 28 days (Vehicle or 28 days of BMX-001). The group that received BMX-001 for 7 days, received subcutaneous injections of vehicle during days 8 to 28. The vehicle group had a slightly slower body weight recovery (**A**). Neurological deficits were significantly improved with the BMX-001 treatment at both day 7 and day 28 post-stroke (**B**). No intra-group difference was found on the infarct volume, although the vehicle group had a larger infarct (**C**). Neurologic scores were expressed as median ± IQR, and other data were expressed as mean ± SD, ** *p* < 0.01.

All the rats recovered from surgery, received treatment, and survived for 28 days. The vehicle rats had a slightly slower body weight recovery (Figure 3A, body weight at post-stroke day 7, 261 ± 47 g in the vehicle group, 276 ± 46 g at 7 days of BMX-001 treatment, and 280 ± 33 g at 28 days of BMX-001 treatment, *p* = 0.37).

BMX-001 treatment promoted post-stroke functional recovery. For rats receiving 7 days of active BMX-001 treatment, neurologic scores were improved on day 7 (Figure 3B, 17 ± 6 in vehicle, 10 ± 8 in BMX-001, *p* = 0.0015) and on day 28 (13 ± 7 in the vehicle, 6 ± 5 in BMX-001, *p* = 0.0013). For rats receiving 28 days of BMX-001 treatment, neurologic scores were improved on day 7 (Figure 3B, 17 ± 6 in vehicle, 11 ± 8 in BMX-001, *p* = 0.0085) and on day 28 (13 ± 7 in the vehicle, 6 ± 4 in BMX-001, *p* = 0.009). The vehicle group had a larger total infarct (Figure 3C, 137 ± 63 mm3 in the vehicle, 131 ± 66 in 7 days of BMX-001, and 112 ± 64 in 28 days of BMX-001, *p* = 0.46). The data supported the conclusion that one week of BMX-001 treatment is sufficient for obtaining long-term protection.

### 3.4. Experiment 4: BMX-001 Improved 28-Day Post-Stroke Functional Deficits in Spontaneous Hypertension Rats

For the 30 young male spontaneous hypertension rats that were subjected to 90 min MCAO, the mean arterial blood pressure was high in both groups before the stroke (127 ± 16 mmHg in the vehicle and 124 ± 21 in BMX-001). As reference, the normal mean arterial blood pressure in young male Wistar rats is around 80 mmHg. Perioperative physiological parameters, including blood pressure, blood gas, glucose, and hematocrit, were comparable in both groups (Table 2).

All of the rats recovered from surgery; however, four rats died after one week (one vehicle rat died 10 days post-stroke; three BMX-001 rats died 8, 9, and 10 days after the stroke, respectively). At the completion of the study, 14 vehicle and 12 BMX-001 rats survived for 28 days.

**Figure 4:**
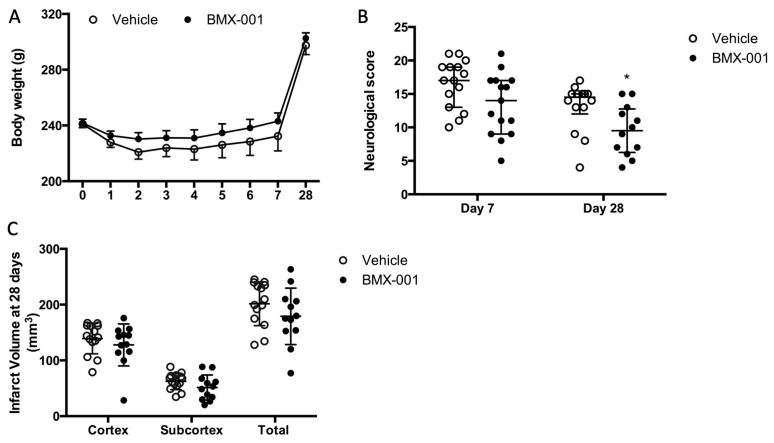
BMX-001 improved 28-day post-stroke functional recovery in young male spontaneous hypertension stroke rats. The animals had a 90 min MCAO and then received an intracarotid bolus infusion of vehicle or BMX-001, followed by subcutaneous maintenance doses for 7 days. The vehicle group had a slightly slower body weight recovery (**A**). Neurological deficits were significantly improved with BMX-001 treatment on day 28 post-stroke (**B**). No intra-group difference was found in infarct volumes, although the vehicle group had a larger infarct (**C**). Neurologic scores were expressed as median ± IQR, and other data were expressed as mean ± SD, * *p* < 0.05.

The vehicle rats had a slow body weight recovery (Figure 4A), similar to the course observed in other experiments. The neurologic score in the vehicle group was 17 ± 6 on day 7 and 14.5 ± 3 on day 28 (Figure 4B). BMX-001 improved neurologic score (14 ± 8 on day 7, *p* = 0.05 vs. vehicle; 9.5 ± 6.25 on day 28, *p* = 0.018 vs. vehicle, Mann–Whitney test). The infarct volumes were smaller in the BMX-001 group (Figure 4C, total infarct volume, 202 ± 39 mm^3^ in vehicle, 179 ± 51 in BMX-001, *p* = 0.21; cortex infarct volume, 139 ± 27 mm3 in vehicle, 128 ± 37 in BMX-001, *p* = 0.37; subcortex infarct volume, 63 ± 15 mm3 in vehicle, 51 ± 23 in BMX-001, *p* = 0.14).

### 3.5. Experiment 5: BMX-001 Improved 28-Day Post-Stroke Outcomes in Aged Female Rats

**Figure 5:**
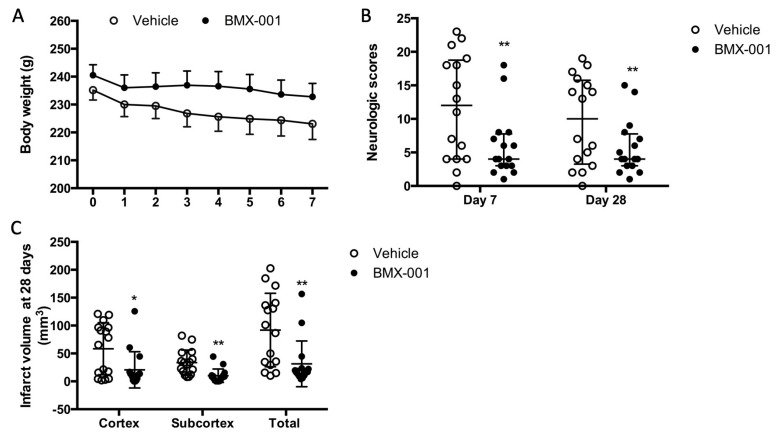
BMX-001 improved 28-day post-stroke outcomes in aged female Fisher 344 rats. Body weight was monitored daily on the first 7 days after the stroke (**A**). Neurologic functions were evaluated on day 7 and day 28 after the stroke (**B**). Infarct volume was measured at 28 days after the stroke (**C**). BMX-001 treatment significantly attenuated neurologic deficits and reduced infarct volume. Neurologic scores were expressed as median ± IQR, and other data were expressed as mean ± SD, * *p* < 0.05, ** *p* < 0.01.

A total of 34 aged rats were subjected to 90 min MCAO, and recovered from surgery. Two rats died at day 2 post-stroke due to a large infarct; these included one vehicle- and one BMX-001-treated rat. The rats were randomly assigned to the treatment groups after surgery. The BMX-001 group had slightly higher blood pressure before stroke (114 ± 11 mmHg) compared to the vehicle group (105 ± 17 mmHg, *p* = 0.06). Other physiological variables were similar. There was a different body weight recovery pattern between aged and young rats. Within the first week post-stroke, all the rats continued to lose body weight, although soft food was provided (Figure 5A). Young rats usually began to gain weight from the third day post-stroke. The body weight was 223 ± 22 g on day 7 post-stroke in the vehicle group, which represented a 12 g loss from the baseline of the body weight (235 ± 14 g). The BMX-001-treated group had a body weight of 233 ± 19 g on day 7 post-stroke, which was an 8 g loss from the baseline (241 ± 15 g). This indicated that the general status was improved by the BMX-001 treatment.

The vehicle rats had more severe neurologic deficits compared to those treated with BMX-001 on both day 7 and day 28 post-stroke (Figure 5B). Neurologic scores in the vehicle group were 12 ± 14.75 on day 7 and 10 ± 12.5 on day 28 compared to 4 ± 4.75 on day 7 and 4 ± 4.75 on day 28 in the BMX-001 group (*p* < 0.001 on day 7 and on day 28 vs. vehicle). The infarct volume was significantly reduced after BMX-001 treatment as well. The total infarct volume was 92 ± 65 mm^3^ in the vehicle-treated rats, including a cortex infarct volume of 58.5 ± 46 mm^3^ and a subcortex infarct volume of 33.5 ± 11 mm^3^ (Figure 5C). The BMX-001 group had a 60% reduction in infarct volume (total infarct volume 31 ± 41 mm^3^, *p* = 0.0037 vs. vehicle; cortex infarct volume 21 ± 32 mm^3^, *p* = 0.0122 vs. vehicle; subcortex infarct volume 10 ± 12 mm^3^, *p* = 0.0010 vs. vehicle).

### 3.6. Experiment 6: BMX-001 Accumulated at the Site of Brain Injury

A total of 4 rats were subjected to a 90 min MCAO, and then 30 µg/kg of BMX-001 was given through the carotid artery immediately following reperfusion. Blood and brains were harvested 30 min later. BMX-001 contents were found in the amounts of 11.3 ± 1.2 nM in plasma samples, 7.4 ± 8.6 nM in the ipsilateral brain hemisphere, and 0.5 ± 0.3 nM in the contralateral brain hemisphere. Direct intracarotid infusion led to a 15-fold greater BMX-001 concentration at the site of brain injury than in the contralateral hemisphere.

### 3.7. Experiment 7: Intracarotid BMX-001 Infusion Did Not Affect the Permeability and Vascular Structure of Blood–Brain Barrier 

**Figure 6:**
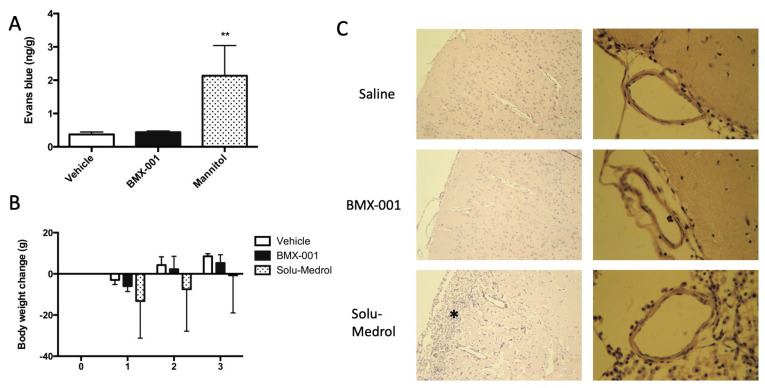
Effect of BMX-001 on the permeability and vascular structure of blood–brain barrier (BBB). Evans blue content was measured in the brain after the intracarotid infusion of vehicle, BMX-001, and Mannitol (positive control) (**A**). BMX-001 did not affect the BBB, and Mannitol did increase BBB permeability. Another set of rats were given vehicle treatment, BMX-001, and Solu-Medrol. Body weight was monitored on days 1, 2, and 3 after intracarotid infusions (**B**). Solu-Medrol caused a sickness in rats and induced brain damage (* represents the damaged area) (**C**). No histologic damage was found in the BMX-001 and vehicle groups. Data were expressed as mean ± SD, ** *p* < 0.01.

After the intracarotid infusion of the vehicle, BMX-001 and mannitol, we injected 0.1 mL 2% Evans blue through the jugular vein in 12 non-stroke rats. Blood was flushed using normal saline 30 min later, and then brains were harvested for Evans blue content measurement. Mannitol served as a positive control, and a significant increase in Evans blue content was found in the Mannitol group (Figure 6A). The BMX-001 group had a slightly high Evans blue content, and an intra-group difference was not detected between the vehicle and BMX-001 groups.

An additional 12 non-stroke rats were used for brain histology. Solu-Medrol served as a positive control. All the rats had a body weight decrease on day 1 after intracarotid infusion (Figure 6B) and then recovered on day 2 and day 3. The Solu-Medrol group did not recover to baseline even after 72 h of infusion. A brain histology demonstrated that there was brain damage in the rat that received Solu-Medrol. Brain tissues and vessels were normal in the rats treated with the vehicle and BMX-001 treatments (Figure 6C).

### 3.8. Experiment 8: No Evidence of an Interaction between BMX-001 and t-PA Was Found

When blood allowed to clot was incubated with saline (vehicle), 1 mg/mL t-PA, or 1 mg/mL t-PA plus 50 µ g/mL BMX-001 at 37 °C for 30 min, blood clot formation was reduced by 0.14 ± 4.05%, 44.77 ± 25.77%, and 43.23 ± 24.88%, respectively. Saline had a negligible effect on blood clots while t-PA or t-PA plus BMX-001 significantly dissolved the blood clot. There was no difference between t-PA and t-PA plus BMX-001.

## 4. Discussion

Extensive clinical trials have proved that endovascular thrombectomy is an effective way to restore the blood flow in selected stroke patients and improve the clinical outcome [10,19,27,28,29,30]. It was reported that endovascular thrombectomy was associated with significantly higher rates of angiographic revascularization at 24 h compared with standard medical care (75.8* vs. 34.1%; OR, 6.49; 95% CI, 4.79–8.79; *p* < 0.001) [27]. In the patients with stroke in the proximal anterior intracranial circulation, thrombectomy with a stent retriever within 6 h after onset plus intravenous t-PA reduced disability at 90 days over the entire range of scores on the modified Rankin scale (*p* < 0.001) [8]. Currently, the time window for thrombectomy has been extended to 24 h [14]. However, some thrombectomy patients did not recover their functional independence, and an exploration of the new therapy adjunct to thrombectomy is needed. In the clinical trials of patients with acute ischemic stroke caused by a proximal intracranial arterial occlusion, intra-arterial treatment with t-PA within 6 h after symptom onset is effective and safe for revascularization [31]. Thus, intra-arterial fibrinolytics are used as adjunct to mechanical thrombectomy to further improve the reperfusion [32,33,34]. We demonstrated that manganese porphyrins provided protection in an animal model of ischemic stroke even when given 6 h after reperfusion [22]. This study was designed to evaluate the potential of BMX-001 as a treatment adjunct to mechanical thrombectomy for improving the outcome of stroke. In the experiments conducted herein, we found that an intracarotid infusion of BMX-001, a compound with sustained redox activity, immediately following reperfusion improved post-stroke neurologic deficits in a rat model of cerebral ischemia where gender, age, and hypertension were accounted for. The largest benefit was observed in aged rats, where the infarct volume was also substantially reduced. We also noted no effects on the vessel wall and no drug interaction with t-PA.

Manganese (III) pentacationic *ortho N*-substituted pyridylporphyrins, or *N*,*N’*-substituted imidazolylporphyrins, are a class of redox-active compounds with exceptional reactivities towards diverse reactive species, including superoxide. In turn, they are commonly regarded as mimics of superoxide dismutases [23,24]. Studies of ours and others provided direct evidence that Mn porphyrins suppress inflammation, which arises as a consequence of the oxidative stress induced by increased levels of reactive species in different tissue injuries, including stroke and renal ischemia/reperfusion injuries [23,24]. Several compounds, including manganese (III) *meso*-tetrakis (*N*-ethylpyridinium-2-yl)porphyrin (MnTE-2-PyP^5+^, AEOL10113, BMX-010), manganese (III) *meso*-tetrakis (*N*,*N’*-diethylimidazolium-2-yl)porphyrin (MnTDE-2-ImP^5+^, AEOL10150), and manganese (III) *meso*-tetrakis (*N*-hexylpyridinium-2-yl)porphyrin (MnTnHex-2-PyP^5+^) improved neurologic and histologic outcomes in rats and mice models of transient focal cerebral ischemia [21,22,35]. A single dose of MnTDE-2-ImP^5+^ provided a dose-dependent improvement in neurologic and histologic outcomes when assessed one-week post-ischemia. However, it failed in the long-term outcome assessment. Nevertheless, the one-week treatment provided a persistent and substantive reduction in both neurologic deficits and infarct volume at 8 weeks post-ischemia [21].

The newest generation of Mn(III) porphyrins, MnTnBuOE-2-PyP^5+^ (Mn (III) *meso*-tetrakis(*N*-(2′-n-butoxyethyl)pyridinium-2-yl)porphyrin, BMX-001), combines superb redox-based potency and lipophilicity with low toxicity [23,24,36]. A wealth of in vitro and in vivo data forwarded BMX-001 into four Phase II clinical trials in cancer patients on the radioprotection and chemoprotection of normal tissue while suppression of cancer [24]. Due to its clinical development, we have selected BMX-001 as a candidate adjunct to thrombectomy. In this stroke study, BMX-001 significantly improved neurologic deficits and reduced infarct size in young male Wistar rats when assessed at 7 days post-stroke. At 28 days post-stroke, the evaluation also demonstrated the protective effect of BMX-001 on neurologic functional improvement in female, SHR, and aged rats. The intracarotid infusion of BMX-001 did not affect BBB permeability and vascular structure. It does not react with t-PA and is therefore safe for use as an adjunct to thrombectomy or a t-PA treatment.

In long-term experiments, at 28 days post-stroke, most of the infarcted tissue was lost and replaced with a cavity filled with fluid in young male, female, and SHR rats. The infarct volume in such experiments was calculated by subtracting the remaining ipsilateral brain tissue from the volume of the contralateral hemisphere. This histologic evaluation is different from the histologic outcome assessed at 7 days post-stroke, which was measured directly from the infarcted area. The post-stroke volume changes in both the ischemic hemisphere and non-ischemic hemisphere impose challenges for long-term histologic assessment. Thus, in future studies, we will add sex- and age-matched naïve controls to assure the accuracy of the evaluation of the tissue damage when the post-stroke events are assessed at later times.

Along with efficacy, we have also performed mechanistic studies to understand the events behind the suppression of inflammation by the class of Mn(III) *ortho N*-substituted pyridylporphyrins. These compounds exhibit their effects via inhibition of NF-κB and activation of Nrf2 transcription factors [23,24,37].

NF-κB is essential in maintaining normal cellular functions, especially in immune responses and tissue repair [38]. However, when the brain experiences a stroke, the NF-κB becomes activated, and this process is accompanied by an upregulation of pro-inflammatory cytokines, adhesion molecules, and proapoptotic processes [39] that further contribute to increased brain damage [40,41]. In a rat stroke study on MnTnHex-2-PyP^5+^, we have demonstrated the inhibition of NF-κB and subsequent downregulation of NF-κB-controlled pro-inflammatory cytokines, TNF-α and IL-6 [22]. Based on our studies, we understand that BMX-001 catalyzes the H_2_O_2_-driven oxidation (*S*-glutathionylation) of the cysteine of the p65 subunit of NF-κB, thus prevents its DNA binding, and in turn the transcription process that would have otherwise induced overwhelming inflammation [23,24]. This process is described in detail in [37].

Nrf2 transcription factor plays a central role in the cellular response to oxidative stress via control of endogenous antioxidant defenses, such as Mn superoxide dismutase, glutathione peroxidase, peroxiredoxins, and heme oxygenase-1, as well as phase II detoxification enzymes such as glutathione *S*-transferase and NAD(P)H;quinone oxidoreductase 1 (NQO1). Two early studies suggested that Mn porphyrin activates Nrf2. In a rat renal ischemia/reperfusion study, the MnSOD, glutathione peroxidase, peroxiredoxins 2, 3, and 5, and thioredoxin reductase 1 were upregulated by MnTnHex-2-PyP^5+^. Consequently, the significant reversal of morphological changes and decrease in the specific ischemic markers lipocalin-2, mucin-1, and galectin-3 were demonstrated [42]. In a mouse stroke study, the protection of mitochondrial enzyme, aconitase, by MnTE-2-PyP^5+^ was seen [43]. The Fe-S clusters of aconitase are susceptible to superoxide-driven damage. Such damage is a specific marker of the increased levels of mitochondrial superoxide and indicates that MnSOD was upregulated, presumably via the Nrf2 pathway [43]. However, direct evidence on Nrf2 activation by BMX-001 was reported in a study on hematopoietic stem/progenitor cells [44]. As a result of Nrf2-signaling activation by BMX-001, endogenous antioxidative defenses such as MnSOD, catalase, glutathione-*S*-transferase, and NQO1 proteins were upregulated. This in turn significantly enhanced the number of hematopoietic stem/progenitor cells [44]. Mechanistically, and similarly to NF-κB, BMX-001 catalyzes the H_2_O_2_-driven oxidation (*S*-glutathionylation) of the cysteine of Keap1 (which is a Nrf2 regulatory protein) [37]. Such oxidation in turn enables Nrf2 to translocate into the nucleus and start transcription. Consequently, endogenous antioxidative proteins are upregulated, offering protection to normal tissues such as the brain during ischemia/reperfusion stroke insult.

## 5. Conclusions

BMX-001 is a Mn porphyrin-based redox-active metal complex currently in several clinical trials. We have demonstrated herein that the intracarotid infusion of BMX-001, immediately following the reperfusion, improved post-stroke neurologic deficits in a rat model of cerebral ischemia regardless of gender, age, and hypertension. Thus, BMX-001 has the potential to further improve the clinical outcomes of stroke patients when used as an adjunct to a thrombectomy.

## Figures and Tables

**Table 1:** Perioperative Physiologic Values (Mean ± SD) in Young Male Wistar Rats.

	Vehicle *n* = 19	7 Days of BMX-001 *n* = 19	28 Days of BMX-001 *n* = 19
10 min before stroke			
MAP (mmHg)	76 ± 7	75 ± 9	75 ± 7
pH	7.476 ± 0.034	7.481 ± 0.033	7.487 ± 0.039
PCO_2_	36 ± 5	36 ± 3	35 ± 4
PO_2_	157 ± 32	168 ± 18	172 ± 13
Glucose	109 ± 17	104 ± 19	110 ± 17
Hct	42.6 ± 1.6	42.6 ± 1.4	42.2 ± 1.3
45 min after stroke onset			
MAP (mmHg)	72 ± 9	69 ± 7	69 ± 7
pH	7.450 ± 0.035	7.462 ± 0.048	7.433 ± 0.049
PCO_2_	38 ± 4	37 ± 6	39 ± 6
PO_2_	162 ± 21	162 ± 17	162 ± 17
10 min post-stroke			
MAP (mmHg)	71 ± 6	67 ± 5	71 ± 6

MAP, mean blood pressure; SD, standard deviation; PCO_2_, partial pressure of carbon dioxide; PO_2,_ partial pressure of oxygen; Hct, hematocrit.

**Table 2:** Perioperative Physiologic Values (Mean ± SD) in Young Male SHR Rats.

	Vehicle *n* = 15	BMX-001 *n* = 15	*p*
10 min before stroke			
MAP (mmHg)	127 ± 16	124 ± 20	0.58
pH	7.402 ± 0.023	7.384 ± 0.046	0.19
PCO_2_	39 ± 3	41 ± 6	0.38
PO_2_	173 ± 24	175 ± 18	0.72
Glucose	97 ± 31	93 ± 30	0.71
Hct	48 ± 3	48 ± 3	0.76
45 min after stroke onset			
MAP (mmHg)	91 ± 13	90 ± 9	0.93
pH	7.379 ± 0.027	7.375 ± 0.038	0.71
PCO_2_	42 ± 4	42 ± 5	0.83
PO_2_	170 ± 16	175 ± 15	0.37
10 min post-stroke			
MAP (mmHg)	89 ± 9	83 ± 11	0.15

SHR, spontaneous hypertension rats; MAP, mean blood pressure; SD, standard deviation; PCO_2_, partial pressure of carbon dioxide; PO_2,_ partial pressure of oxygen; Hct, hematocrit.

## Data Availability

Full data are available from the corresponding author upon reasonable request.
